# Untargeted Metabolomics Identify a Panel of Urinary Biomarkers for the Diagnosis of Urothelial Carcinoma of the Bladder, as Compared to Urolithiasis with or without Urinary Tract Infection in Dogs

**DOI:** 10.3390/metabo12030200

**Published:** 2022-02-24

**Authors:** Maria Malvina Tsamouri, Blythe P. Durbin-Johnson, William T. N. Culp, Carrie A. Palm, Mamta Parikh, Michael S. Kent, Paramita M. Ghosh

**Affiliations:** 1 Veterans Affairs-Northern California Health System, Mather, CA 95655, USA; 2 Department of Urologic Surgery, School of Medicine, University of California Davis, Sacramento, CA 95718, USA; 3 Department of Public Health Sciences, University of California Davis, Davis, CA 95616, USA; bpdurbin@ucdavis.edu; 4 Department of Surgical and Radiological Sciences, School of Veterinary Medicine, University of California Davis, Davis, CA 95616, USA; wculp@ucdavis.edu (W.T.N.C.); mskent@ucdavis.edu (M.S.K.); 5 Department of Medicine and Epidemiology, School of Veterinary Medicine, University of California Davis, Davis, CA 95616, USA; cpalm@ucdavis.edu; 6 Division of Hematology and Oncology, Department of Internal Medicine, School of Medicine, University of California Davis, Sacramento, CA 95718, USA; mbparikh@ucdavis.edu; 7 Department of Biochemistry and Molecular Medicine, School of Medicine, University of California Davis, Sacramento, CA 95718, USA

**Keywords:** canine urothelial carcinoma, urinary metabolomics, molecular and comparative oncology

## Abstract

Urothelial carcinoma (UC), the most common urologic cancer in dogs, is often diagnosed late because the clinical signs are shared by other non-malignant lower urinary tract disorders (LUTD). The urine-based BRAF^V595E^ test for UC is highly effective only in certain breeds; hence additional non-invasive biomarkers of UC are needed. Here, urine from dogs with UC (*n* = 27), urolithiasis (*n* = 8), or urolithiasis with urinary tract infection (UTI) (*n* = 8) were subjected to untargeted metabolomics analyses, using GC-TOF-MS for primary metabolites, QTOF-MS for complex lipids, and HILIC-QTOF MS for secondary and charged metabolites. After adjusting for age and sex, we identified 1123 known metabolites that were differentially expressed between UC and LUTD. Twenty-seven metabolites were significant (1.5 ≤ log_2_FC ≤ −1.5, adjusted *p*-value < 0.05); however, 10 of these could be attributed to treatment-related changes. Of the remaining 17, 6 (hippuric acid, N-Acetylphenylalanine, sarcosine, octanoylcarnitine, N-alpha-methylhistamine, glycerol-3-galactoside) discriminated between UC and LUTD (area under the ROC curve > 0.85). Of the 6 metabolites, only hippuric acid and N-alpha-methylhistamine were discriminatory in both male (*n* = 20) and female (*n* = 23) dogs, while sarcosine was an effective discriminator in several breeds, but only in females. Further investigation of these metabolites is warranted for potential use as non-invasive diagnostic biomarkers of dogs with UC that present with LUTD-related clinical signs.

## 1. Introduction

Urothelial carcinoma (UC) is the most common type of urinary bladder malignancy in dogs, affecting >50,000 dogs annually in the US. Reported breed predilections include Scottish Terriers, Shetland sheepdogs, wirehair fox terriers, West Highland white terriers, and beagles. Middle-aged or elderly female dogs are most commonly affected [1]. Early diagnosis of this disease is often challenging due to non-specific clinical signs, such as stranguria, pollakiuria, and hematuria as well as secondary bacterial infection. These clinical signs are shared by other lower urinary tract disorders (LUTD) such as urinary tract infection (UTI) and urolithiasis [1].

The current mechanisms of detection of UC involve cytopathology, histopathology, and a recently developed urine test to identify BRAF^V595E^ mutations, which are present in ~65–80% of canine UC [2,3]. Since then, many studies have been undertaken to identify additional biomarkers that distinguish canine UC from other diseases, but few, if any, are used clinically. We previously evaluated the expression levels of 5 miRNA associated with UC pathophysiology (miR-34a, let-7c, miR-16, miR-103b, and miR-106b) in the blood and urine of dogs with UC vs. LUTD [4]. These studies indicated a need for unbiased identification of non-invasive markers that distinguish between UC and other LUTD.

The urine is a rich source of metabolites, and multiple studies have evaluated the metabolites present in the urine of dogs with UC. Previous studies have demonstrated differential expression of various metabolites (urea, choline, methylguanidine, citrate, acetone, β-hydroxybutyrate) in the urine of dogs with UC compared to those of healthy dogs [5]. However, a comparison between dogs with UC and other LUTD has not been conducted. Dysuria (straining to urinate), pollakiuria (increase in urination frequency), and haematuria (bloody urine) are also primary signs of canine bladder stones (urocystoliths), with or without secondary UTI. However, there is little evidence of markers that can distinguish between the two. In this study, therefore, we embark on an investigation to differentiate between bladder stones and bladder cancer via urine metabolomics.

Altered metabolic regulation of tumor cells has been recognized as one of the hallmarks of cancer [6]. Metabolomics profile the end-products of gene expression and cell regulatory processes, while also reporting on environmental effects. The cancer microenvironment itself is the richest source of metabolic biomarkers that can report on early and successive changes in metabolism as cancer progresses, integrating the effects of genetic and environmental factors [7]. Metabolomics can be used for the identification of cancer risk factors, the characterization of the tumorigenic potential of certain metabolic pathways, and the discovery of potential diagnostic cancer biomarkers [8].

In this study, we sought to identify a unique set of metabolic markers that can accurately diagnose UC. Previous studies have identified differentially present metabolites in dogs with UC and clinically normal dogs [5,9,10]. Here we hypothesize that differentially present metabolites could distinguish between UC and other LUTD in dogs (especially urolithiasis) that are evaluated for lower urinary tract-related clinical signs. We identify six metabolites (hippuric acid, N-Acetylphenylalanine, sarcosine, octanoylcarnitine, N-α-methylhistamine (NAMH), glycerol-3-galactoside) in the urine of subjects with UC that are distinctively different from those with urolithiasis, with or without UTI (used as the control group). However, only two—hippuric acid and NAMH—could distinguish between UC and control in both males and females, and they were effective mostly in retrievers and in all other breeds, but not in terriers. We also determined that neither urethral obstruction, nor other factors such as spaying/neutering, or the use of antibiotics or NSAID, or the development of metastasis or chemotherapy, affected the expression of these metabolites.

## 2. Results

### 2.1. Subject Characteristics

The clinicopathological characteristics of the dogs that participated in this study are shown in Table 1. Forty-three dogs from multiple breeds were recruited, including terriers (12/43, 27.9%), retrievers (6/43, 14%), and shepherds (5/27, 11.6%). In total, 27 had UC, 8 had urolithiasis only, and 8 had both UTI and urolithiasis. We did not collect urine from any dog with UTI alone, nor did we collect urine from any female dogs with urolithiasis. Of the 43 dogs, 20 (46.5%) were female and 23 (53.5%) were male; however, in the UC group, 15 (55.6%) of the animals were female whereas 12 (44.6%) were male (*p* = 0.009). Median age of the dogs with UC was 11 years (range 6–14 years), whereas dogs with urolithiasis (6.5 years (range 1–13 years)), or UTI + urolithiasis (7 years (range 0.3–12 years)) were significantly younger (*p* < 0.001).

In the UC group, more than 90% of the animals were castrated/spayed, with only one female (1/15) and one male (1/12) being intact. Similarly, in the control group, 1/5 female and 3/11 male dogs were intact. Several of the dogs presented with urinary obstructions (7/43(16.3%)), including 5/27(18.5%) in the UC group; 3/27 (11.1%) of dogs with UC had already developed metastases at the time of urine collection. Some dogs were actively receiving non-steroidal anti-inflammatory drugs (NSAIDS) (15/43 (34.9%)), including (12/27, 44.4%) in the UC group; 8/43 (18.6%) were also receiving antibiotics, including 7 (25.9%) with UC at the time of urine collection. The others diagnosed with a UTI went on to receive antibiotics after urine was already collected. Of the dogs with UC, only a few had received chemotherapeutics (4/27, 14.8%) at the time urine was collected. However, there was no statistical difference between the dogs with UC vs. those with urolithiasis with or without UTI, with respect to any of these factors.

### 2.2. Differentially Expressed Metabolites in UC vs. Control Reflects Changes in Medication and Diet, as Well as Internal Genetic Alterations Due to Disease Progression

**Figure 1:**
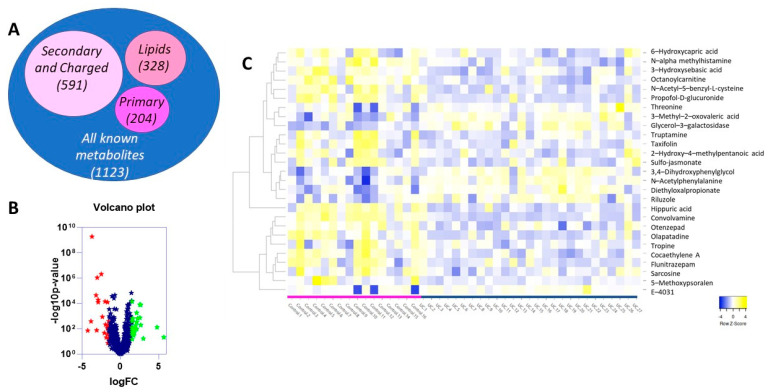
Identification of 17 metabolites differentially expressed between urine of dogs with UC vs. control. (**A**) Nested Venn Diagram depicting the different types of metabolites identified as differentially expressed between the urine of dogs with urothelial carcinoma and control dogs. (**B**) Volcano plot depicting differentially expressed metabolites between UC and control groups. Red stars represent significantly downregulated metabolites (logFC ≤ −1.5 and adjusted *p*-value < 0.05) whereas green stars represent significantly upregulated metabolites (logFC ≥ 1.5 and adjusted *p*-value < 0.05). (**C**) Heatmap showing 17 differentially expressed metabolites in UC (right) vs. control (left) groups. These represent the final 17 metabolites after those related to treatment were removed. Complete Linkage was used as the clustering method with Spearman Rank correlation as a distance measurement method.

Untargeted metabolomic analysis was conducted for the abovementioned 43 urine samples and normalized to creatinine levels as described in Section 4. Because age and sex were significantly different between UC vs. the urolithiasis and UTI + urolithiasis groups combined (referred herein as the control group), data were adjusted for age and sex. Untargeted analyses identified 6981 compounds (Figure 1A) differentially expressed between the two groups, of which 1123 corresponded to known metabolites (Figure 1B). This included 204 primary metabolites, 328 lipids, and 591 secondary and charged metabolites. Only the known metabolites were analyzed further. Of the 1123 known metabolites, only 27 metabolites (Figure 1C) were considered significant (−1.5 ≥ log_2_ fold change ≥ 1.5, adjusted *p*-value < 0.05); 2 primary metabolites and 25 secondary bioamines (Table 2). Of the 27, 8 were upregulated, including one primary metabolite; while 19 were downregulated, including one primary metabolite.

Several of the differentially identified metabolites are considered products of drug metabolism (Table S1). Those downregulated in UCs included: Olapatadine, a metabolite of antihistamines [11]; propofol-β-D-glucuronide, a metabolite of propofol (Pubchem, [12]); Taxifolin (dihydroquercetin), a flavonoid used as an antioxidant in nutritional supplements [13]; Otenzepad, a muscarinic M2 receptor antagonist [14]; 5-methoxypsoralen, an anti-psoriatic agent [15]; and convolvamine, a tropane alkaloid with anxiolytic, anti-depressant, and sedative activity [16]. Metabolites upregulated in UC included E-4031, an experimental class III antiarrhythmic drug [17], Riluzole, a glutamate blocker to treat amyotrophic lateral sclerosis [18], Cocaethylene, formed by the simultaneous administration of ethanol and cocaine [19], and Flunitrazepam, a benzodiazepine used to treat severe insomnia and assist with anesthesia [20]. These drugs were likely received as anesthetic or sedation protocols or used to treat their current or concurrent diseases.

### 2.3. Identification of Six Metabolites That Are Distinctly Expressed in the Urine of Dogs with Urothelial Carcinoma vs. Urolithiasis with or without a UTI

**Figure 2:**
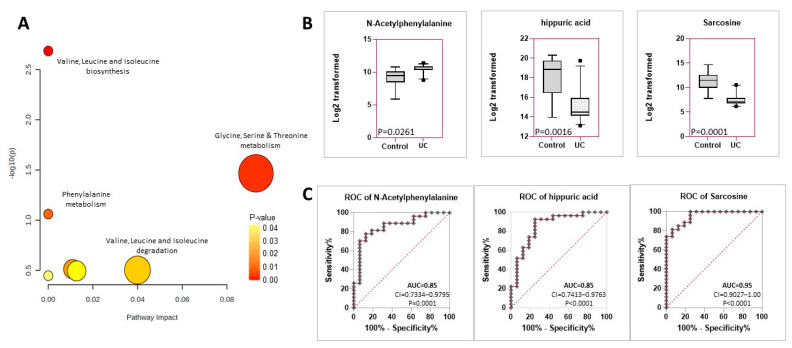
Pathway analysis revealing differentially expressed pathways between urine of dogs with UC vs. control. (**A**) KEGG Pathway Analysis in canine UC as compared to the control group based on the 27 initial metabolites identified as differentially expressed between UC and controls. The color intensity indicates the *p*–value (indicated in inset), whereas the size of each circle indicates the number of metabolites included in the pathway. (**B**) Box plots showing the differential expression of components of the phenylalanine metabolism pathway (N-Acetylphenylalanine (Afalanine); Hippuric Acid) and the Glycine, Serine, and Threonine Metabolism pathways (sarcosine). All *p*–values are adjusted *p*-values. (**C**) ROC curves for the above metabolites. Area under the ROC curve (AUC) is calculated as % sensitivity vs. 100-% specificity. AUC > 0.85 is highly discriminatory.

KEGG pathway analysis was performed to identify the metabolic pathways that are differentially expressed in dogs with UC as compared with the control group. The analysis was performed using differentially expressed metabolites after adjusting for age and sex (Figure 2A). Various pathways were shown to be possibly affected, and the metabolites in each pathway are presented in Table 3. Valine, leucine, and isoleucine biosynthesis pathway (*p* = 0.00103); glycine, serine and threonine metabolism (*p* = 0.0178); and phenylalanine metabolism (*p* = 0.0629) were the most enriched pathways in UC vs. control groups (Figure S1)

We next determined whether specific components of the pathways distinguished between UC and controls. A noteworthy component of the phenylalanine metabolism pathway is hippuric acid, which is significantly decreased in urine from dogs with UC compared to controls (log_2_FC = −3.55, adjusted *p*-value = 0.0016) (Figure 2B, Table 2). This metabolite is an acyl glycine produced by the conjugation of glycine and benzoic acid, found normally in the urine as a metabolite of aromatic compounds. Similarly, N-Acetylphenylalanine (Afalanine), also a member of this pathway, was significantly increased in urine from dogs with UC compared to control animals (log_2_FC = +1.6295, adjusted *p*-value = 0.0261) (Figure 2B). Other components of this pathway include 5-Methoxypsoralen (commonly upregulated in psoriasis) and Taxifolin (an antioxidant in nutritional supplements), both downregulated in UC but not included in this list as they are likely components of ingested drugs/food supplements.

We next investigated whether components of the phenylalanine metabolism pathway can distinguish between the UC and control groups. The area under the Receiver Operating Characteristics (ROC) curve (AUC) for hippuric acid and NAMH both showed specificity and sensitivity with AUC = 0.85 for both (*p* = 0.0001 or <0.0001, respectively) (Figure 2C). In contrast, members of the valine, leucine, and isoleucine biosynthesis pathway (L-Threonine, 3-Methyl-2-Oxovaleric acid, 2-hydroxy-4-methylpentanoic acid), although individually differentially expressed between the two groups, did not show a significant ability to distinguish between UC and control (AUC < 0.75) (Figure S2). However, sarcosine, which mapped to the Glycine, Serine and Threonine Metabolism pathway, was also a significant distinguishing factor (AUC = 0.95, *p* < 0.0001) (Figure 2C) (threonine (Figure S2) is also a component of this pathway). Taken together, alterations of the phenylalanine pathway and the glycine, serine and threonine metabolism pathway were distinctive in UC vs. controls.

**Figure 3:**
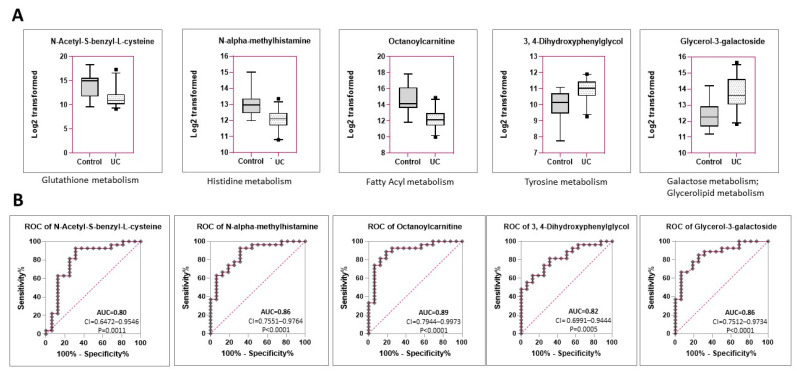
Three additional metabolites, not in the above pathways, were also differentially expressed, and could distinguish between UC and control. (**A**) Box plots showing the differential expression of additional metabolites significantly different between UC and controls of which three are downregulated (N-Acetyl-S-benzyl-L-cysteine, N-alpha-methylhistamine, and Octanoylcarnitine) and two are upregulated (3,4-Dihydroxyphenylglycol and glycerol-3-galactoside). Related pathways are listed below the plot. (**B**) ROC curves for the above metabolites. Area under the ROC curve (AUC) is calculated as sensitivity percentage vs. 100-specificity percentage. AUC > 0.85 is considered to be highly discriminatory. Thus, only three of the five metabolites were considered highly discriminatory (Octanoylcarnitine, N-alpha-methylhistamine, and glycerol-3-galactoside).

Other metabolites differentially expressed in UC vs. control include N-Acetyl-S-benzyl-L-cysteine (log_2_FC = −3.88, adjusted *p*-value = 0.0063), NAMH (log_2_FC = −1.57, adjusted *p*-value = 0.0084), and Octanoylcarnitine (log_2_FC = −2.2, adj.*p*.val. = 0.011), all downregulated in UC compared to controls (Figure 3A, Table 2). On the other hand, 3,4-dihydroxyphenylglycol (DHPG, log_2_FC = +1.585, adjusted *p*-value = 0.0111) and glycerol-3-galactoside (log_2_FC = +1.672, adjusted *p*-value = 0.0478) were increased in UC compared to controls. Of the above metabolites, the fatty acyl octanoylcarnitine showed the highest ability to distinguish between dogs with UC and control (AUC = 0.8958 (CI:0.7944–0.9973)) (Figure 3B); however, two others were also highly discriminatory (AUC > 0.85), including NAMH, a product of histidine metabolism (AUC = 0.8657, CI:0.7551–0.9764), and glycerol-3-galactoside, a component of both the galactose and the glycerolipid metabolism pathways (AUC = 0.8623, CI = 0.7512–0.9734). Other metabolites tested (Figure S3) failed to sufficiently discriminate between UC vs. control (Figure S4) despite being differentially expressed between the groups. Taken together, these results identify 6 urine-based metabolites (hippuric acid, N-Acetylphenylalanine, sarcosine, octanoylcarnitine, NAMH, glycerol-3-galactoside) that can likely distinguish between UC vs. control groups.

### 2.4. Hippuric Acid and NAMH Are Discriminatory between UC vs. Control in Both Male and Female Dogs

**Figure 4:**
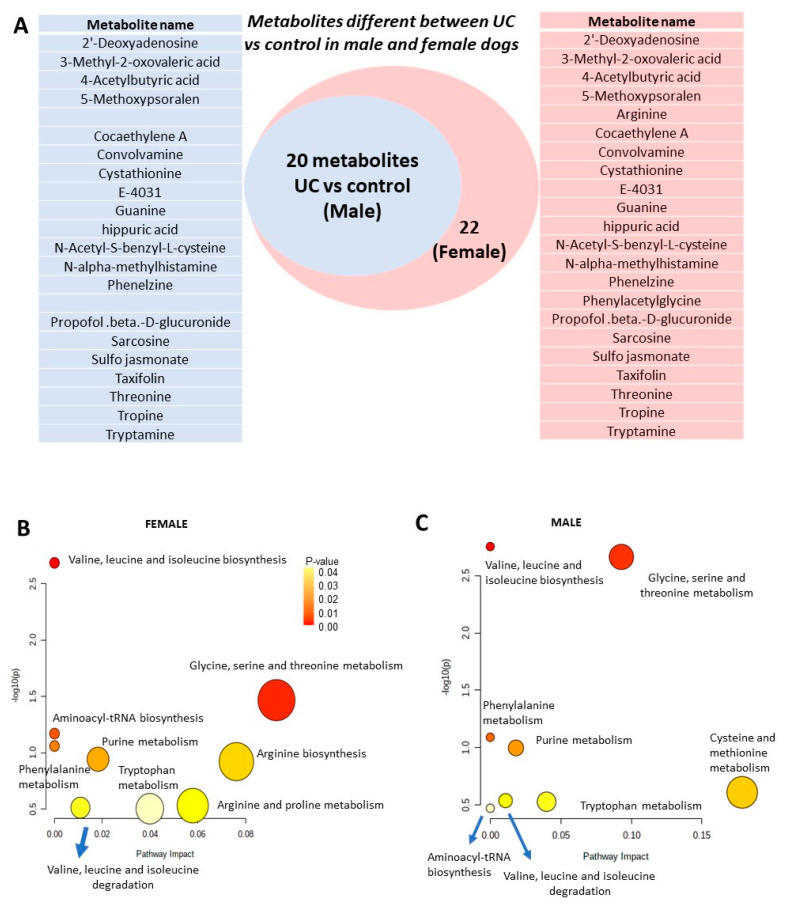
(**A**) Venn diagram demonstrating differentially expressed urine metabolites in UC vs. controls among male and female dogs. Differentially expressed metabolites in each group are outlined in the inset accompanying each group. (**B**) KEGG Pathway Analysis in canine UC as compared to the control group in female dogs only. The color intensity indicates the *p*-value (indicated in inset), whereas the size of each circle indicates the number of metabolites included in the pathway. (**C**) KEGG Pathway Analysis in canine UC as compared to the control group in male dogs only. The color intensity indicates the *p*-value (indicated in inset), whereas the size of each circle indicates the number of metabolites included in the pathway.

UC in dogs has a higher prevalence in female than male dogs (~1.8:1 ratio) [21]. We therefore compared the metabolites of male dogs (*n* = 12) vs. those of female dogs (*n* = 15). Evaluation of the urine of female dogs with UC (*n* = 15) and comparing them to the urine of control female dogs (*n* = 5) identified 22 differentially expressed metabolites (Table S2). Of these, 14 metabolites have been reported as differentially expressed in Table 2, including three of the highly discriminatory metabolites (hippuric acid, NAMH, sarcosine). Male dogs showed a similar differentiation pattern; however, arginine and phenylacetylglycine were differentially expressed in UC vs. control in females but not in males (Figure 4A). The urine of female dogs with UC appeared to be richer in metabolites, with a larger number of pathways represented (Figure 4B) compared to those of male dogs with UC (Figure 4C).

**Figure 5:**
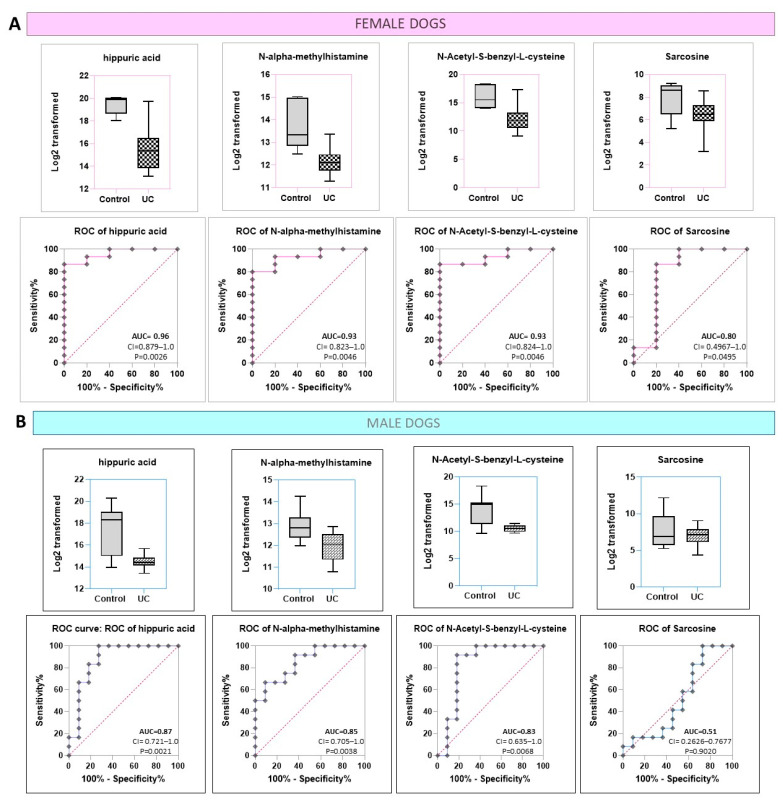
Comparison of the expression of specific metabolites in male and female dogs with UC vs. controls. (**A**) Box plots showing the differential expression of four metabolites significantly different between UC vs. control (hippuric acid, N-Acetyl-S-benzyl-L-cysteine, N-alpha-methylhistamine, and sarcosine) in female dogs. The lower level shows ROC curves for the above metabolites. Only three of the four metabolites were considered highly discriminatory (hippuric acid, N-Acetyl-S-benzyl-L-cysteine, N-alpha-methylhistamine) (AUC > 0.85). (**B**) Box plots showing the differential expression of four metabolites significantly different between UC vs. control in male dogs. The lower level shows ROC curves for the above metabolites. Only two of the four metabolites were considered highly discriminatory (hippuric acid, N-alpha-methylhistamine) (AUC > 0.85).

Arginine biosynthesis and arginine and proline metabolism were highly enriched in female dogs but not in males. On the other hand, cysteine and methionine metabolism was enriched in male dogs but not in females. In both females and males, hippuric acid, NAMH, N-Acetyl-S-benzyl-L-cysteine, and sarcosine were differentially expressed between UC and control (Figure 5). However, analysis of the ROC curve for these conditions showed that only three (hippuric acid, NAMH, N-Acetyl-S-benzyl-L-cysteine) were highly discriminatory in females with UC as compared to controls (AUC > 0.85) (Figure 5A). In contrast, in males, only hippuric acid and NAMH were discriminatory (AUC > 0.85) (Figure 5B). Of the rest, some, such as N-Acetylphenylalanine, were significantly discriminatory in females but not in males, whereas others, such as glycerol-3-galactoside and octanoylcarnitine, were discriminatory in males but not in females (Figure S5). Therefore, of the six metabolites identified above, only hippuric acid and NAMH showed promise as markers of UC in both sexes.

### 2.5. Breed-Related Differences in Metabolite Expression Show That Changes in Metabolites Observed Are Mostly Driven by Retrievers and Not Terriers

We next investigated whether the differential expression of the 6 metabolites that showed significant ability to discriminate between UC and control (hippuric acid, N-Acetylphenylalanine, sarcosine, octanoylcarnitine, NAMH, glycerol-3-galactoside) was driven by specific breeds. Table 4 shows the different breeds used in this study classified as defined by the American Kennel Club (AKC) and the United Kennel Club (UKC). None of the dogs in the Herding and Hound groups in this study experienced urolithiasis. Hence, we were not able to compare the expression of these metabolites between UC and control within these groups. The Companion breed group was represented by a single animal in the urolithiasis arm, while the Non-Sporting and Working Groups were represented by only one animal each in the UC arm; hence, we could not compare metabolites in UC vs. control in these groups either. The group with the largest number of animals, both in the UC and the control arms, were the terriers and retrievers (Table 4), hence we further investigated the contribution of these groups in the overall differential expression of metabolites.

**Figure 6:**
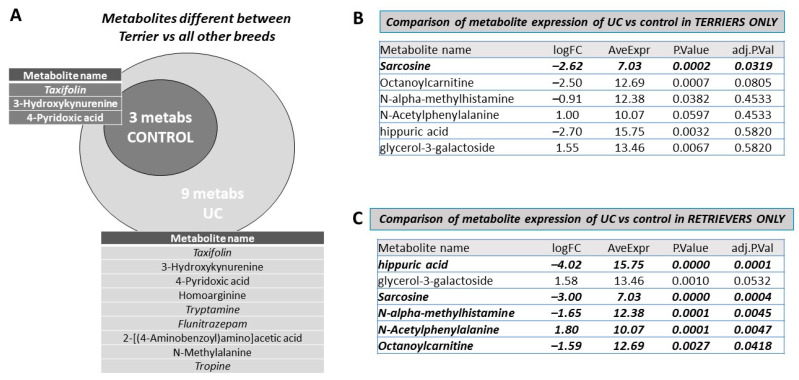
Breed specific differences between UC vs. control. (**A**) Venn diagram demonstrating differentially expressed metabolites in terrier breeds vs. all other breeds among dogs with UC only and comparing it with differentially expressed metabolites in terriers and all other breeds among control dogs. Differentially expressed metabolites in each group are outlined in the inset accompanying each group. (**B**) Differential expression between UC vs. control of the six metabolites in terrier breeds only. Note that, of the six metabolites deemed highly discriminatory in Figure 2 and Figure 3 (highlighted in bold), only sarcosine was differentially expressed in terrier breeds. (**C**) Differential expression between UC and controls of the six metabolites in retriever breeds only. Note that, of the six metabolites deemed highly discriminatory in Figure 2 and Figure 3, five were differentially expressed in retriever breeds. Glycerol-3-galactoside was not differentially expressed in either terrier breeds or in retriever breed groupings.

To establish baselines, we first investigated whether there were any basic differences between terriers and non-terriers with respect to any metabolite. Neither the primary metabolites nor any of the lipids showed any significant differences between the terrier and non-terrier groupings (not shown). However, among biogenic amines, there were significant differences between terrier vs. non-terrier groups in both the UC and the control groups (Table 5). Of these, three (Taxifolin (up); 3-Hydroxykynurenine (down) and 4-Pyridoxic acid (down)) were differentially expressed in terriers compared to non-terrier groups in both the UC and controls, suggesting that these differences were inherent in terriers. However, six other metabolites were differentially expressed in the terrier group in the UC patients examined. Five were upregulated, including tryptamine, tropine, Homoarginine, Flunitrazepam, and N-Methylalanine, while 2-[(4-Aminobenzoyl)amino]acetic acid was down regulated (Figure 6A). In contrast to the terrier group, there were no UC-exclusive differences between those in the retriever and non-retriever groupings (Table S3).

Finally, we investigated whether the six metabolites that were distinctive in UC vs. controls were more discriminatory in terrier vs. retriever groups. Figure 6B demonstrates that, in terriers, of the 6 metabolites mentioned above, only sarcosine (log_2_FC = −2.62, adjusted *p*-value = 0.032) was highly discriminatory between UC and controls. In contrast, Figure 6C demonstrates that, in the retriever grouping, 5 of the 6 metabolites mentioned above, namely N-Acetylphenylalanine (log_2_FC = 1.799, adjusted *p*-value = 0.0047), N-acetyl-S-benzyl-L cysteine (log_2_FC = −4.468, adjusted *p*-value = 0.000293), Octanoylcarnitine (log_2_FC = −1.593, adjusted *p*-value = 0.041), sarcosine (log_2_FC = −2.99, adjusted *p*-value = 0.000355), NAMH (log_2_FC = −1.647, adjusted *p*-value = 0.00452), and hippuric acid (log_2_FC = −4.022, adjusted *p*-value = 5.25 × 10^−5^), were highly discriminatory. Thus, of the 6 metabolites, only sarcosine showed differential expression in both the terrier and retriever groups. Other metabolites differentially expressed between UC and controls in the terrier and retriever groups are listed in Tables S4 and S5, respectively. When all other breeds were considered, the differential expression of these five metabolites between UC and controls remained statistically significant (Table S5). These results demonstrate that while hippuric acid and NAMH, which were highly distinctive in both male and female dogs between UC and control groups, are discriminatory in the retriever breed grouping and in other breeds, they are not distinctive for the terrier group.

### 2.6. The Difference in These Metabolites Is Not Affected by Either NSAID or Antibiotic Use, or by Urethral Obstruction

Urethral obstruction was reported in 5 dogs with UC and 2 dogs with urolithiasis (Table 1). Comparison of these dogs in the urethral obstruction group identified 37 metabolites of which only 12 were unique to the urethral obstruction group, while 25 were common with the overall group identified in Table 2 (Table 6). This included all 6 metabolites identified in Figure 2 and Figure 3 as being discriminatory in UC vs. urolithiasis/UTI. Hence, we investigated whether these metabolites are differentially expressed between dogs with urethral obstruction (*n* = 5) vs. those with no urethral obstruction (*n* = 22) in the UC group only. This comparison yielded only stearic acid (log_2_FC = 4.24, adjusted *p*-value = 0.0067) and palmitic acid (logFC = 4.21, adjusted *p*-value = 0.0067) as being differentially expressed between the groups (not shown). Thus, both dogs with urethral obstruction and those with no urethral obstruction showed similar discriminatory behavior of the six selected metabolites—hence, urethral obstruction is unlikely to be the cause of the differences seen between the UC and urolithiasis/UTI groups observed in either Table 2 or Table 6. In contrast, nine other metabolites showed significant differential expression between UC vs. control only in subjects experiencing urethral obstruction (2,3-Dihydroxybenzoic acid B, D-Glucosamine, Haloperidol, Metanephrine, Palatinose, and four phospholipids), while four metabolites showed significant differential expression between UC vs. control only in subjects experiencing *no* urethral obstruction (2′-Deoxyadenosine, 3,4-Dihydroxyphenylglycol, Phenylacetylglycine and Riluzole) (Table 6).

Finally, we observed that in the dogs with UC, 7 received antibiotics and 12 received NSAIDs at the time of urine collection. Therefore, we investigated whether these factors affected the expression of the metabolites differentially expressed in Table 2. However, among those with UC, only piroxicam (the NSAID most commonly used in dogs with UC) was differentially expressed in the urine of those taking NSAID and those not taking an NSAID.

Among dogs with UC, although 13 metabolites were differentially expressed in those taking antibiotics and those not taking antibiotics (Table S6), none coincided with the list of metabolites differentially expressed between the UC and control groups. Because only one dog within the control group was on antibiotics at the time of urine collection, we could not compare metabolite expression between the UC and control groups within the antibiotic use group. Interestingly, most of the metabolites identified as differentially expressed in the antibiotics group, among the ones within the UC group, were identified as either bacterial products (vanillic acid, adipic acid) or food metabolites, perhaps as part of antibiotic administration. Therefore, the six discriminatory metabolites are not likely to be caused by treatment with antibiotics or NSAIDs. Similarly, analyses demonstrated that these changes are not affected by the presence of metastases or by the concurrent use of chemotherapy (data not shown). These results indicate that the six metabolites identified above may be truly inherent to the tumor and not affected by treatments or other conditions.

## 3. Discussion

Clinically, dogs with UC and those with urocystoliths or UTI present with very similar clinical signs, including stranguria, pollakiuria, and hematuria, as well as secondary bacterial infection [1], and it is often difficult to differentially diagnose between malignancy and other non-malignant diseases of the lower urinary tract. While a clinical test is available for the detection of UC in the urine, namely the BRAF^V595E^ mutation analysis, this test was found to be more effective in terriers (73% carry the mutation), than in other breeds (36% carry the mutation) (*p* < 0.05) [22]. In the present study, we used urine from dogs of multiple breeds to determine whether subjects with UC may be distinguished from those with urocystoliths with or without UTI (combined in this study as the control group) prior to expensive imaging studies. We identify six metabolites (hippuric acid, N-Acetylphenylalanine, sarcosine, octanoylcarnitine, NAMH, glycerol-3-galactoside) in the urine of subjects with UC as compared to the control group. However, further analyses showed that not all six can be used in both female and male dogs; in fact, of the six, only two—hippuric acid and NAMH (both significantly downregulated in UC)—are able to distinguish between UC and controls in both males and females. Additionally, these two metabolites were distinctly different in UC vs. controls, mostly in retrievers and in all other breeds, whereas they were not discriminatory between the two groups in terriers. On the other hand, sarcosine discriminated between UC and controls in all breeds, but only in female dogs. We also determined that neither urethral obstruction, nor other factors such as spaying/neutering, or the use of antibiotics or NSAID, or the development of metastasis or chemotherapy, affected the expression of these six metabolites.

Hippuric acid is a prominent member of the phenylalanine metabolism pathway and is produced downstream of L-phenylalanine [23]. Urinary hippuric acid has been previously thought to be a harbinger of environmental toxic solvent exposures; however, recent data indicate that hippuric acid is excreted daily in the urine, even in the absence of organic solvent exposure [24]. Amino acid catabolism is a crucial component for cancer cell proliferation and progression, as it is involved in various processes such as nucleotide synthesis, lipogenesis, metastasis, epigenetic modifications, and evasion of tumor recognition by the immune system [25]. Phenylalanine is one of the essential amino acids, and the importance of phenylalanine metabolism has been identified in several human cancers, including colorectal cancer [26] and oral squamous cell [27] and non-small cell lung carcinoma, whereas phenylalanine restriction was associated with decreased tumor invasion in melanoma [28]. This study identified N-Acetylphenylalanine, an N-acetylated form of phenylalanine (Pubchem, [12]), to be significantly upregulated in the UC group, indicating a probable role of this metabolite in canine UC. On the other hand, hippuric acid, an intermediate of phenylalanine metabolism, was strongly downregulated in the UC group as compared to the control group. Hippuric acid has been shown to be significantly downregulated in the urine of human patients with non-muscle invasive [29,30] and, very recently, high-grade bladder cancer (BlCa) [31]. It is important to note that decreases in hippuric acid levels have also been seen in conjunction with aging [32], because dogs with UC were in general older than controls, although age and sex differences were accounted for in the biostatistical analysis described. Hippuric acid is also a major regulator of calcium oxalate lithiasis [33], the most common type of stones in urolithiasis, and high levels of hippuric acid was thought to dissolve calcium oxalate stones. Therefore, loss of hippuric acid in UC is not simply a case of lack of this metabolite in patients with stones. Loss of hippuric acid has also been associated with frailty and changes in gut microflora [32]—possibly due to lack of adequate nutrition associated with the onset of carcinoma. A major cause of concern is the lack of hippuric acid excretion, which could also indicate its accumulation in the kidney, or an inability to metabolize phenylalanine. Thus, we believe that loss of urinary hippuric acid would be a strong candidate for an indicator of UC development in dogs.

The glycine, serine, and threonine metabolic pathway was also found to be significantly upregulated in the urine of dogs with UC vs. the control group. Glycine is used for the biosynthesis of glutathione, the main antioxidant used to scavenge reactive-oxygen species (ROS) produced by cancer cells and maintain redox homeostasis. Serine also contributes to ROS elimination, either by direct conversion to glycine by the enzyme serine hydroxymethyltransferase or by fueling the folate cycle with the side production of NADPH [25,34]. Sarcosine (also called *N*-methyl-glycine) is a methylated form of glycine, and threonine can be also be converted to glycine. Threonine was significantly upregulated in UC compared to controls (FC = 1.8; adjusted *p*-value = 0.0378), while sarcosine was downregulated (FC = −3.34; adjusted *p*-value = 0.0001), indicating the accumulation of threonine in UC patients that is not converted to glycine. However, of the two, only loss of sarcosine showed a sufficient discriminatory ability to discriminate between the two conditions (AUC = 0.95) and was included in the final panel of six metabolites. It is important to note that of all the metabolites, sarcosine was the only one that showed discriminatory properties in all dog breeds; however, its discrimination ability is only confined to female dogs. Increased sarcosine urinary levels in humans with prostate cancer have the potential as a diagnostic biomarker [35]. It is curious as to why sarcosine is not discriminatory between UC and controls in males as well—although it is downregulated in UC in males as well as in females. Similar to hippuric acid, sarcosine is also downregulated with age in humans, and increases with dietary restrictions [36]. It is to be noted that sarcosine is an ingredient of toothpaste and is thought to fight cavities, and is also used for treating mental health disorders, including schizophrenia and depression [37]. Similar to glycine, sarcosine also activates the N-methyl-D-aspartate (NMDA) receptor and blocks the GlyT1 (type 1 glycine) transporter. Significantly, the efficacy of sarcosine was enhanced in female schizophrenic patients compared to males [38], suggesting a female-specific mechanism.

The other significant metabolite downregulated in UC is NAMH, a histamine agonist selective for the receptor subtype H3 that lowers blood pressure and heart rate. NAMH is reportedly promoted in Helicobacter pylori-infected human gastric mucosa, and was suggested to act directly on histamine H(2) receptors (H2Rs) as well as to stimulate acid secretion [39]. It is also used to treat migraines in humans [40]. NAMH has previously been shown to play a role in bladder cancer. In a study of non-muscle invasive bladder cancer (NMIBC) in human patients, a disease not usually seen in dogs, urinary NAMH levels were seen to increase with treatment with intravesical Bacillus Calmette-Guérin (BCG), the most effective therapy for high-grade NMIBC, and decrease when the treatment was attenuated [41]. Further, urinary NAMH levels increased after BCG immunotherapy in patients who responded, compared to those who did not respond [41]. While most canine UC is muscle invasive, the above adds further evidence that urinary NAMH levels are low in untreated bladder cancer.

Of the three other metabolites that were found to be discriminatory between UC and control when all animals were considered, N-Acetyl-S-benzyl-L-cysteine (Benzylmercapturic acid) is a metabolite of toluene that is used in the diagnosis of toluene exposure. Toluene has been proposed as a marker of UTI that can be used by an electronic nose for diagnosis of this disease [42]; hence it is likely increased in UTI and not decreased in UC. Next, lipid remodeling is one of the hallmarks of cancer, and lipids have been recognized to regulate multiple other hallmarks of cancer as well, by providing the energy required to sustain tumor proliferation, migration, and invasion while evading cell death by immune recognition, tumor suppressors, or apoptotic mechanisms [43]. Even though lipids can be obtained either from the diet or be synthesized by other substrates, cancer cells largely rely on de novo fatty acid synthesis [44]. The Glycerolipid/Free Fatty acid (GL/FFA) cycle, the interface between synthesis of GL from FFA and glycerol and GL hydrolysis for the production of FFA, plays an important role in cancer, especially during cancer-associated cachexia (e.g., a state of nutrient deprivation) [45]. Glycerol-3-galactoside, a possible metabolite of glycerolipid synthesis, was increased in UC versus the control group, probably indicating a role for this pathway in canine UC. Finally, octanoylcarnitine forms when carnitine is covalently linked with the medium-chain fatty acid, octanoic acid [46], and can be oxidized through mitochondrial β-oxidation for the production of acetyl-coA [47]. Acetyl-coA can then be used for the production of FFA, fueling the TCA cycle [48] or for epigenetic modifications through the addition of acetyl groups [6]. Octanoylcarnitine was significantly downregulated in dogs with UC as compared with the control group, as has been shown in the blood of women with breast cancer [49], potentially indicating the increased demand of this metabolites in tumor cells. Other fatty acyls that were differentially expressed in UC vs. control included 6-hydroxycaproic acid and 3-hydroxysebacic acid, but these metabolites lacked sufficient discriminatory power to distinguish between the two groups.

One of the most important results of this study is that while all six metabolites, except glycerol-3-galactoside, were discriminatory in the retriever group and in other breeds, only sarcosine was discriminatory in the terrier group. This is important because the BRAF^V595E^ mutation is more prevalent in terriers with UC than in other breeds with UC. Therefore, it is important to identify other putative agents that in the future can be developed into markers of UC in other breeds, perhaps in combination with BRAF. It is obvious from the results that terriers are different from other breeds, not only with respect to the presence of the BRAF mutation in UC, but also in other respects—such as the inability of 5 of the 6 metabolites to discriminate between UC and controls in these breeds.

As mentioned above, all 6 metabolites were differentially present in the retriever breed grouping with UC vs. controls, but only one (sarcosine) was discriminatory in terriers. However, the term “terrier” represents a group of breeds that are somewhat heterogeneous. Furthermore, only hippuric acid and NAMH were statistically significant when we stratified the analysis to females and males. This could be related to the smaller sample size when we stratified the samples for the different comparisons. Thus, hippuric acid and NAMH can be tested further as markers of UC in both male and female dogs of various species, including retriever breeds, but is not likely to be discriminating in terrier breeds. Further studies should be conducted in a larger cohort of dogs of multiple breeds of both sexes, to further characterize potential breed-related and sex-related differences in biomarker expression and UC pathophysiology. A limitation of the current study is that the results from the discovery cohort were used to construct the ROC curves. The use of additional cohorts to test the markers identified will correct this drawback.

## 4. Materials and Methods

### 4.1. Urine Collection and Handling

Urine collection was approved by UC Davis Institutional Animal Care and Use Committee (IACUC) and clinical trials review boards, under the protocol # 20416. All dogs participating in the study were privately owned, screened, and recruited at the UC Davis Veterinary Medical Teaching Hospital (VMTH) between 2011 and 2020, with informed owner consent. A sample of 1 mL of urine was collected from patients with confirmed UC (*n* = 27), urolithiasis (*n* = 8), or UTI + urolithiasis (*n* = 8). Inclusion criteria for dogs with UC were defined as the presence of naturally occurring UC of the urinary bladder and/or urethra that had been previously confirmed through histopathology or cytologically by a board-certified veterinary pathologist and retrieved from the medical records of the patient. Exclusion criteria included lack of definite diagnosis of UC, incomplete medical history, and presence of another malignancy of the urinary tract. Inclusion criteria for the control group included the confirmation of urolithiasis through urinalysis and UTI by bacterial culture (urolithiasis and UTI group) or urolithiasis with a negative urinary culture (urolithiasis group). Exclusion criteria included the presence of a malignancy of the urinary tract. For all dogs, age, weight, breed, castration status, and medical history were recorded. Urine samples were centrifuged (5 min, 3000 RPM, 4 °C), the supernatant was collected, filtered through a 0.22 μm filter (Millipore Sigma SLGVM33RS Medical Millex^®^ GV Sterile Syringe Filter, Merck, Temecula, CA, USA), and stored at −80 °C until further analysis.

### 4.2. Urinary Creatinine Levels

Ten microliters of urine was used to assess urinary creatinine levels using the Creatinine Parameter Assay Kit (R&D Systems, cat.# KGE005), according to manufacturer’s instructions. Curve expert Professional software (v2.6.5) was used for the generation of standard curves and calculation of creatinine levels.

### 4.3. Metabolomic Analyses

For the initial metabolomic analysis, urine samples were normalized to creatinine levels, and 0.5 mL of urine from each dog was submitted to the West Coast Metabolomics Center, UC Davis [50,51]. The samples were spiked with a recovery standard cocktail, extracted in methanol, and dried under vacuum to remove organic solvents. The extracts were separated into three fractions and characterized using three independent platforms, as described below. The reproducibility of the extraction protocol was assessed by the recovery of the xenobiotic compounds spiked in every urine sample prior to extraction:

1. Primary metabolites: Using derivatization and GC-TOF MS, primary metabolites such as amino acids, sugars, and hydroxyl acids were profiled. The BinBase database used has over 1200 primary metabolites and the ability to annotate unknown metabolites [52].
2. Complex lipids: >350 lipids covering 12 lipid classes (e.g., ceramides, cholesteryl esters, free fatty acids, phosphatidyl-cholines, -serines and -ethanolamines, lysophospholipids, sphingomyelins, and triacylglycerols) were quantified from an acetonitrile/isopropanol gradient on a charged surface hybrid (CSH) LC column and positive and negative electrospray ionization QTOF MS. The LipidBLAST library has 200,000 MS/MS lipid spectra.
3. Secondary and charged metabolites: Charged, complex hydrophilic compounds such as cholines, nucleotides, nucleosides, methylated, and acetylated amines were measured using HILC QTOF MS; >100 secondary compounds were screened using the standards on this platform. Exposomes, such as metabolites from pharmaceutical drugs, were detected through untargeted analysis.

Data from all three platforms were integrated in a single report to provide an overview of the metabolic status of each dog.

### 4.4. Statistical Analyses

R package limma v.3.46.0 was used for the statistical analyses of the metabolomics raw data in this study, incorporating variance weights from the limma function vooma. Analyses were adjusted for age and sex by including these as covariates in models. For the assessment of differentially expressed metabolites between the groups, data were log_2_ transformed and normalized using cyclic loess normalization. Metabolites were considered statistically significant when the adjusted *p*-value was ≤0.05 and log_2_fold change was ≥1.5 or ≤−1.5. Heatmapper [53] was used to generate a heatmap of differentially expressed metabolites, using Complete Linkage as the clustering method with Spearman Rank correlation as a distance measurement method. Pathway and Enrichment analyses were performed using MetaboAnalyst [54] 5.0 using the Homo sapiens Kyoto Encyclopedia of Genes and Genomes (KEGG) metabolic pathway database. The hypergeometric test was used as an Over-Representation Analysis, and relative betweenness centrality was the measure for Pathway Topology Analysis. GraphPad Prism v9.2.0 was used to construct box plots, volcano plots, and receiver operating statistics (ROC) curves for in-sample predictions. The Human Metabolome Database [55] was used to delineate the function of various metabolites.

## Figures and Tables

**Table 1:** Subject characteristics by group. All comparisons shown are between Urinary Carcinoma (UC) (*n* = 27) and controls (urolithiasis *n* = 8, Urinary Tract Infection (UTI) + urolithiasis, *n* = 8). Age is significantly different between groups (*p* < 0.001), with an older mean age in the UC group compared to the other two groups. The distribution of sexes also differs significantly between groups (*p* = 0.00921), with only male dogs in this study having urolithiasis.

	UC (*n* = 27)	Urolithiasis Only (*n* = 8)	UTI and Urolithiasis (*n* = 8)	All Subjects (*n* = 43)	*p*-Value ^†^
Age					<0.001
Mean (SD)	10.9 (1.9)	7.2 (3.9)	6.2 (4.6)	9.3 (3.5)	
Median (Range)	11 (6–14)	6.5 (1–13)	7 (0.3–12)	10 (0.3–14)	
Gender					0.00921
Female	15 (55.6%)	0	5 (62.5%)	20 (46.5%)	
Male	12 (44.4%)	8 (100%)	3 (37.5%)	23 (53.5%)	
Breed					
Terrier	5 (18.5%)	4 (50%)	3 (37.5%)	12 (27.9%)	0.20218
Shepherd	5 (18.5%)	0	0	5 (11.6%)	0.34365
Retriever	5 (18.5%)	0	1 (12.5%)	6 (14%)	0.60082
Others	12 (44.4%)	4 (50%)	4 (50%)	20 (46.5%)	
Conditions					
Castrated/Spayed	25 (92.6%)	6 (75%)	6 (75%)	37 (86%)	0.17918
Urethral obstruction	5 (18.5%)	1 (12.5%)	1 (12.5%)	7 (16.3%)	1.00000
Presence of metastasis	3 (11.1%)	0	0	3 (7%)	
Treatments					
Antibiotics	7 (25.9%)	0	1 (12.5%)	8 (18.6%)	0.30230
NSAIDS	12 (44.4%)	3 (37.5%)	0	15 (34.9%)	0.07110
Chemotherapy	4 (14.8%)	0	0	4 (9.3%)	

^†^*p*-value for age is from an ANOVA F-test and *p*-values for categorical variables are from Fisher’s Exact Tests.

**Table 2:** Metabolites that are differentially expressed between Urinary Carcinoma and control groups, after adjusting for age and sex (adjusted *p*-value < 0.05; −1.5 ≥ logFC ≥ 1.5). Highlighted in grey are compounds that are probably metabolites of ingested drugs these dogs had received as part of their anesthetic or sedation protocols, or drugs being used to treat their current or concurrent diseases.

Platform	Metabolite Name	logFC	AveExpr	*p* Value	adj.*p*.Val.
Primary metabolites (GCTOF MS)	glycerol-3-galactoside	1.6722	13.4648	0.0006	0.0478
	hippuric acid	−3.5546	15.755	<0.0001	0.0016
Secondary and charged metabolites (HILIC-QTOF MS)	E-4031	4.4226	6.8263	0.0014	0.0314
	Riluzole	2.3073	10.9453	0.0013	0.0305
	Diethyloxalpropionate	1.8298	9.8314	0.0018	0.0387
	3-Methyl-2-oxovaleric acid	1.8166	11.3829	<0.0001	0.0022
	Threonine	1.8112	11.5928	0.0017	0.0378
	N-Acetylphenylalanine	1.6295	10.0706	0.001	0.0261
	3,4-Dihydroxyphenylglycol	1.585	10.7024	0.0002	0.0111
	N-alpha-methylhistamine	−1.5784	12.378	0.0001	0.0084
	3-Hydroxysebacic acid	−1.8382	11.246	0.0007	0.0217
	5-Methoxypsoralen	−1.9007	6.5929	0.0009	0.026
	6-Hydroxycaproic acid	−2.1693	12.4207	0.0005	0.0194
	Octanoylcarnitine	−2.2014	12.6927	0.0002	0.0111
	Otenzepad	−2.2737	8.5251	0.0007	0.0217
	2-Hydroxy-4-methylpentanoic acid	−2.3568	9.71	0.0005	0.0194
	Taxifolin	−2.3879	9.4181	0.0004	0.0186
	Sulfo jasmonate	−2.6733	10.5836	0.0008	0.0253
	Tryptamine	−2.8279	9.4239	0.0001	0.0097
	Flunitrazepam	−2.9725	7.2548	0.0002	0.0097
	Sarcosine	−3.3428	7.0257	<0.0001	0.0001
	Cocaethylene A	−3.3942	7.7349	<0.0001	0.0002
	Propofol. beta.-D-glucuronide	−3.6922	11.0599	0.0002	0.0097
	N-Acetyl-S-benzyl-L-cysteine	−3.8876	12.156	0.0001	0.0063
	Olopatadine	−3.8901	8.6456	0.0002	0.0097
	Convolvamine	−4.0141	8.3401	<0.0001	0.0001
	Tropine	−4.1392	9.54	0.0001	0.0084

After eliminating the drug-related products, 17 metabolites were found to be differentially expressed, likely due to internal genetic or tumor microenvironmental changes associated with disease progression (Table S1), with 11 downregulated and 6 upregulated in UC compared to controls.

**Table 3:** KEGG Pathway analysis in canine Urinary Carcinoma as compared to the control group. Number refers to the number of the metabolites in this study out of all the metabolites of the pathway in the parentheses. FDR: False Discovery rate.

Pathway Name	Metabolites	*p* Value
Valine, Leucine and Isoleucine Biosynthesis	L-Threonine, 3-Methyl-2-Oxovaleric acid, 2-hydroxy-4-methylpentanoic acid	0.00103
Glycine, Serine and Threonine Metabolism	Sarcosine, L-Threonine	0.00178
Phenylalanine Metabolism	Hippuric acid, N-Acetylphenylalanine, 5-Methoxypsoralen, Taxifolin	0.00629
Valine, Leucine, and Isoleucine Degradation	3-Methyl-2-Oxovaleric acid, L-Threonine	0.231
Tryptophan Metabolism	Tryptamine	0.236
Tyrosine Metabolism	3-4-Dihydroxyphenylglycol	0.241
Aminoacyl-tRNA Biosynthesis	L-Threonine	0.271

**Table 4:** Breed variations in Urinary Carcinoma and urolithiasis/urinary tract (U/UTI) infection.

Group	Breed	Defined by	UC (*n* = 27)	U/UTI (*n* = 16)
Herding	Australian Cattle Dog ^†^	AKC	1	0
	Australian Shepherd ^†^	AKC	2	0
	Border Collie ^†^	AKC	1	0
	Collie Mix	AKC	1	0
	German Shepherd	AKC	1	0
	Shepherd Mix	AKC	2	0
	Shetland Sheepdog ^†^	AKC	1	0
Hound	Beagle ^†^	AKC	1	0
	Dachshund	AKC	1	0
	Rhodesian Ridgeback	AKC	1	0
Non-sporting	Bichon Frise ^†^	AKC	0	2
	Dalmatian	AKC	1	0
	French Bulldog	AKC	0	1
Sporting	Brittany/Spaniel mix	AKC	0	1
	Golden Retriever	AKC	2	1
	Labrador Retriever	AKC	2	0
	Labrador Retriever Mix	AKC	1	0
Terrier	American Pit Bull Terrier	UKC	0	1
	Miniature Schnauzer	AKC	1	2
	Terrier Mix	AKC	1	3
	West Highland White Terrier ^†^	AKC	3	1
	Yorkshire Terrier	AKC	0	1
Companion	Chihuahua mix	AKC	0	1
	Miniature Pinscher	AKC	2	0
	Pomeranian	AKC	1	0
Working	Great Pyrenees	AKC	0	1
	Mastiff	AKC	1	0
	Rottweiler	AKC	0	1

^†^ Considered to be high-risk for UC.

**Table 5:** Differences in metabolites from the urine of terrier breeds and non-terrier breeds in the Urinary Carcinoma (UC) arm and control arm.

Terrier vs. Non-Terrier								
	UC				Control			
Metabolite Name	logFC	AveExpr	*p* Value	adj.*p*.Val.	logFC	AveExpr	*p* Value	adj.*p*.Val.
Taxifolin	3.32344	9.4180	6.57 × 10^−5^	0.0191	3.2452	9.4180	0.0001	0.0376
3-Hydroxykynurenine	−7.8520	13.8756	7.22 × 10^−5^	0.0191	−7.7438	13.8756	0.0001	0.0376
4-Pyridoxic acid	−2.2640	12.0144	9.73 × 10^−5^	0.0191	−2.2118	12.0144	0.0001	0.0376
Homoarginine	2.4386	8.33970	0.0002	0.0358				
Tryptamine	3.2555	9.4239	0.0003	0.0359				
Flunitrazepam	3.3556	7.2548	0.0004	0.0359				
2-[(4-Aminobenzoyl) amino]acetic acid	−2.4797	13.9770	0.0004	0.0359				
N-Methylalanine	3.35947	10.8914	0.0006	0.0461				
Tropine	4.2490	9.5399	0.0007	0.0468				

**Table 6:** Differentially expressed metabolites in urine of dogs with urethral obstruction suffering from Urinary Carcinoma (UC) vs. control dogs (log FC ≥ 1.5 and adj. *p*-value ≤ 0.05). Bolded are the ones that were highly discriminatory between UC and control.

		Urethral Obstruction			No Urethral Obstruction		
Metabolite Name	AveExpr	logFC	*p* Value	adj.*p*.Val	logFC	*p* Value	adj.*p*.Val
2,3-Dihydroxybenzoic acid B	10.63	2.59	0.0031	0.0473			
2’-Deoxyadenosine	9.38				−1.52	0.0017	0.0341
2-Hydroxy-4-methylpentanoic acid	9.71	−2.11	0.0015	0.0319	−2.45	0.0007	0.0218
3,4-Dihydroxyphenylglycol	10.70				1.61	0.0004	0.0160
3-Hydroxysebacic acid	11.25	−1.92	0.0001	0.0064	−2.08	0.0001	0.0057
3-Methyl-2-oxovaleric acid	11.38	1.82	0.0000	0.0005	1.90	0.0000	0.0008
4-Acetylbutyric acid	13.37	2.38	0.0009	0.0271	2.33	0.0022	0.0422
5-Methoxypsoralen	6.59	−1.83	0.0010	0.0271	−2.05	0.0007	0.0218
6-Hydroxycaproic acid	12.42	−1.91	0.0015	0.0319	−2.24	0.0006	0.0218
Cer d36:1	9.78	−2.09	0.0005	0.0472			
Cer d42:2 Isomer A	10.95	−1.64	0.0006	0.0472			
Cer-NS d34:1; Cer-NS d18:1/16:0;	8.82	−3.07	0.0005	0.0472			
Cocaethylene A	7.73	−3.19	0.0000	0.0005	−3.45	0.0000	0.0006
Convolvamine	8.34	−4.17	0.0000	0.0000	−4.15	0.0000	0.0000
D-Glucosamine	9.50	−1.63	0.0025	0.0424			
Diethyloxalpropionate	9.83	1.88	0.0010	0.0271	1.99	0.0011	0.0255
E-4031	6.83	4.48	0.0012	0.0294	4.70	0.0013	0.0307
FA 24:1 (nervonic acid)	10.66	1.72	0.0004	0.0472			
Flunitrazepam	7.25	−2.98	0.0001	0.0064	−3.24	0.0001	0.0072
**glycerol-3-galactoside**	**13.46**	**1.69**	**0.0003**	**0.0296**	**1.81**	**0.0003**	**0.0299**
Haloperidol	7.69	−3.14	0.0026	0.0424			
**hippuric acid**	**15.75**	**−3.68**	**0.0000**	**0.0003**	**−3.72**	**0.0000**	**0.0009**
Lobelanidine	9.60	−1.52	0.0016	0.0321	−1.69	0.0011	0.0255
Metanephrine	10.03	−1.62	0.0021	0.0393			
**N-Acetylphenylalanine**	**10.07**	**1.72**	**0.0003**	**0.0138**	**1.77**	**0.0005**	**0.0196**
N-Acetyl-S-benzyl-L-cysteine	12.16	−3.96	0.0000	0.0020	−4.12	0.0000	0.0031
**N-alpha-methylhistamine**	**12.38**	**−1.52**	**0.0001**	**0.0064**	**−1.54**	**0.0003**	**0.0111**
N-Methylalanine	10.89	−2.15	0.0019	0.0376	−2.27	0.0022	0.0422
**Octanoylcarnitine**	**12.69**	**−2.22**	**0.0001**	**0.0064**	**−2.32**	**0.0002**	**0.0077**
Olopatadine	8.65	−3.95	0.0001	0.0064	−4.35	0.0001	0.0047
Otenzepad	8.53	−2.22	0.0007	0.0227	−2.33	0.0008	0.0225
Palatinose	11.85	3.38	0.0023	0.0418			
Phenylacetylglycine	12.62				1.50	0.0008	0.0225
Propofol. beta.-D-glucuronide	11.06	−4.03	0.0000	0.0010	−4.24	0.0000	0.0014
Riluzole	10.95				2.25	0.0026	0.0487
**Sarcosine**	**7.03**	**−3.22**	**0.0000**	**0.0001**	**−3.46**	**0.0000**	**0.0001**
Sulfo jasmonate	10.58	−2.64	0.0008	0.0246	−2.81	0.0008	0.0225
Taxifolin	9.42	−2.49	0.0001	0.0064	−2.68	0.0001	0.0072
Tropine	9.54	−4.28	0.0000	0.0012	−4.41	0.0000	0.0021
Tryptamine	9.42	−2.74	0.0002	0.0069	−2.92	0.0002	0.0077

## Data Availability

All relevant available data is presented in Supplementary Materials.

## Supplementary Materials

The following supporting information can be downloaded a: https://www.mdpi.com/article/10.3390/metabo12030200/s1, Figure S1: KEGG Pathway Analysis in canine UC (*n* = 27) as compared to the control group (*n* = 16) based on 1123 known metabolites identified as differentially expressed between urothelial carcinoma and the control group, Figure S2: Box plots showing the differential expression of components of the Valine, leucine and isoleucine biosynthesis pathway, Figure S3: Box plots showing the differential expression of components of various pathways, Figure S4: ROC curves for the metabolites shown in Supplementary Figure S3, Table S1: Functional and pathway analysis of metabolites that are differentially expressed between Urothelial Carcinoma and Control groups, after adjusting for age and sex, Table S2: Comparison of primary metabolites and biological amines from the urine of male and female dogs that show significant differences in the Urothelial Carcinoma vs. Control arms, Table S3: Differences in metabolites from the urine of retriever vs. non-retriever in the Urothelial Carcinoma and Control arms, Table S4: Comparison of primary metabolites and biological amines from the urine of terriers only that show significant differences in the Urothelial Carcinoma (UC) vs. Control arms, Table S5: Comparison of primary metabolites and biological amines from the urine of retrievers only that show significant differences in the Urothelial Carcinoma (UC) vs. Control arms, Table S6: Comparison of primary metabolites and biological amines from the urine of dogs with Urothelial Carcinoma who had been exposed to antibiotics prior to urine collection vs. those who had not.
