# Supplementary material for: Untargeted Metabolomics Identify a Panel of Urinary Biomarkers for the Diagnosis of Urothelial Carcinoma of the Bladder, as Compared to Urolithiasis with or without Urinary Tract Infection in Dogs
Source: Metabolites. 2022 Feb 24;12(3):200. doi: 10.3390/metabo12030200 (PMC8951005; doi:10.3390/metabo12030200)
Supplement: Supplementary file 1 [file metabolites-12-00200-s001.zip › metabolites-1577821-supplementary.pdf]

SUPPLEMENTARY DATA

Untargeted metabolomics identify a panel of urinary biomarkers for the diagnosis of urothelial carcinoma of the bladder, as compared to urolithiasis, with or without urinary tract infection in dogs

Maria Malvina Tsamouri[1,2*], Blythe P. Durbin-Johnson[3], William T. N. Culp[4], Carrie A. Palm[5], Mamta Parikh[6], Michael S. Kent[4], Paramita M. Ghosh[1,2,7*]

[1]Veterans Affairs-Northern California Health System, Mather, CA, 95655, USA; mtsamouri@ucdavis.edu (MMT)

[2]Department of Urologic Surgery, School of Medicine, University of California Davis, Sacramento, CA, 95718, USA; paghosh@ucdavis.edu (PMG)

[3]Department of Public Health Sciences, University of California Davis, Davis, CA, 95616, USA; bpdurbin@ucdavis.edu (BPDJ)

[4]Department of Surgical and Radiological Sciences, School of Veterinary Medicine, University of California Davis, Davis, CA, 95616, USA; wculp@ucdavis.edu (WTNC), mskent@ucdavis.edu (MSK)

[5] Department of Medicine and Epidemiology, School of Veterinary Medicine, University of California Davis, Davis, CA, 95616, USA; cpalm@ucdavis.edu

[6]Division of Hematology and Oncology, Department of Internal Medicine, School of Medicine, University of California Davis, Sacramento, CA, 95718, USA; mbparikh@ucdavis.edu

[7] Department of Biochemistry and Molecular Medicine, School of Medicine, University of California Davis, Sacramento, CA, 95718, USA

*Correspondence: *PMG; paghosh@ucdavis.edu ; Tel.: +1-916-843-9336; MMT; mtsamouri@ucdavis.edu; Tel.: +1-530-760-5297*

Table of Content

Supplementary Figure S1 Page 2
Supplementary Figure S2 Page 2
Supplementary Figure S3 Page 3
Supplementary Figure S4 Page 4

Supplementary Table S1 Page 6
Supplementary Table S2 Page 8
Supplementary Table S3 Page 9
Supplementary Table S4 Page 9
Supplementary Table S5 Page 10
Supplementary Table S6 Page 12

Supplementary Figure S1

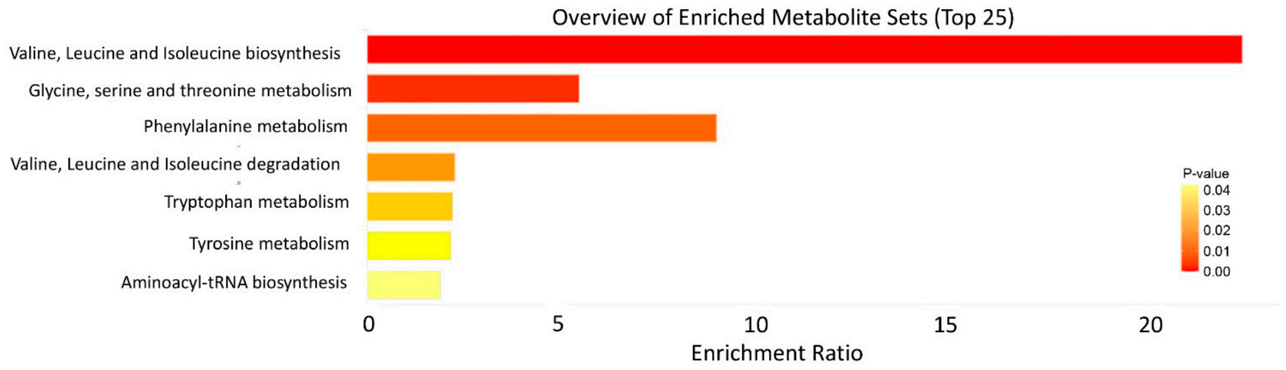

KEGG Pathway Analysis in canine UC (n=27) as compared to the control group (n=16) based on 1123 known metabolites identified as differentially expressed between urothelial carcinoma and the control group. The color intensity indicates the p-value (indicated in inset) whereas the size of each bar indicates the number of metabolites included in the pathway.

Supplementary Figure S2

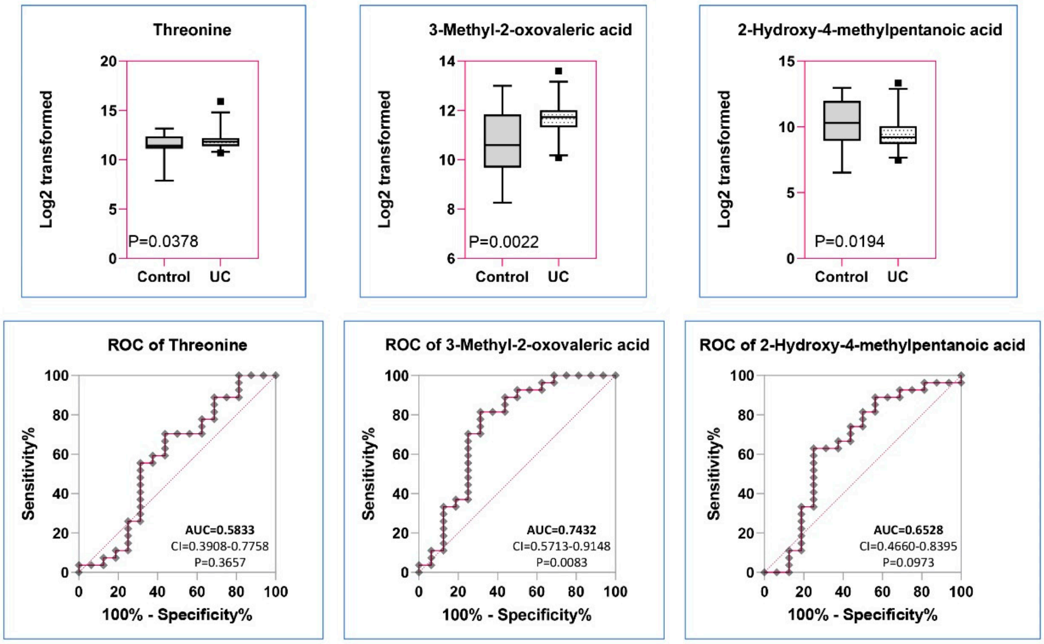

Box plots showing the differential expression of components of the Valine, leucine and isoleucine biosynthesis pathway .Threonine, 3-Methyl-2-oxovaleric acid, 2-hydroxy-4-methylpentanoic acid ***(Upper Row)****. Note that threonine is also a prominent component of the glycine, serine and threonine metabolism pathway as well. All p-values noted are adjusted p-values.* ***(Lower Row)*** *ROC curves for the above metabolites. Area under the ROC curve (AUC) is calculated as % sensitivity vs 100-% specificity. AUC>0.85 is considered to be highly discriminatory. Note that although the three metabolites are differentially expressed in control vs UC, they were unable to discriminate between the two conditions.*

Supplementary Figure S3

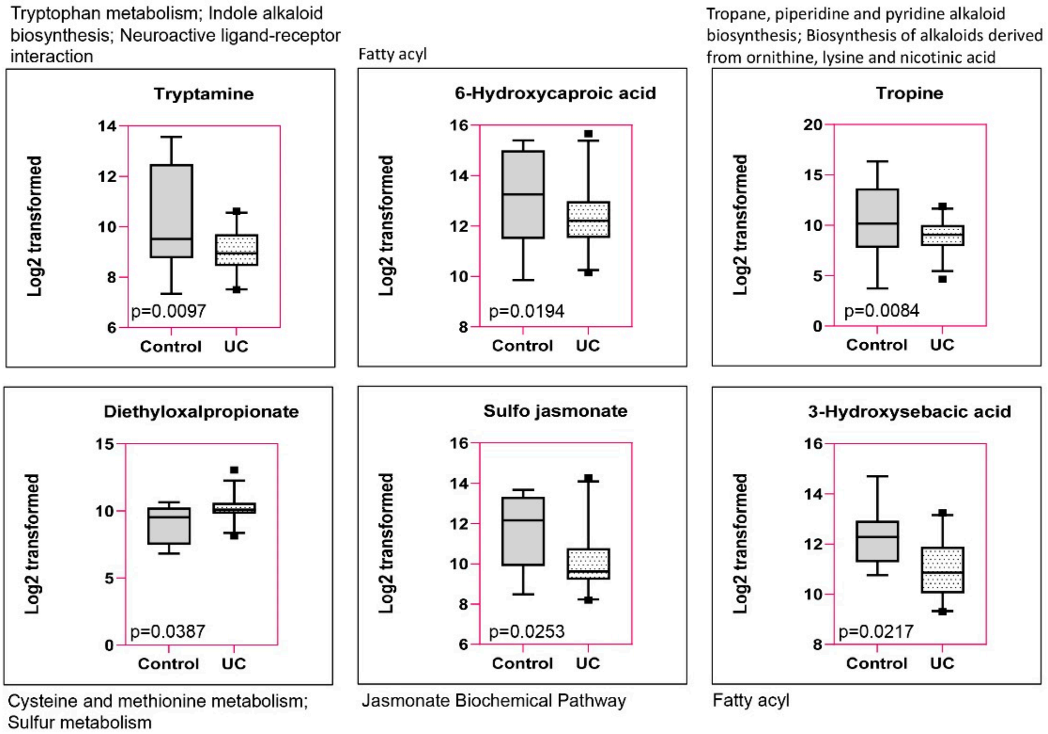

Box plots showing the differential expression of components of various pathways. All p-values noted are adjusted p-values. The pathways in which these metabolites are functional are noted at the top or bottom of the plots.

Supplementary Figure S4

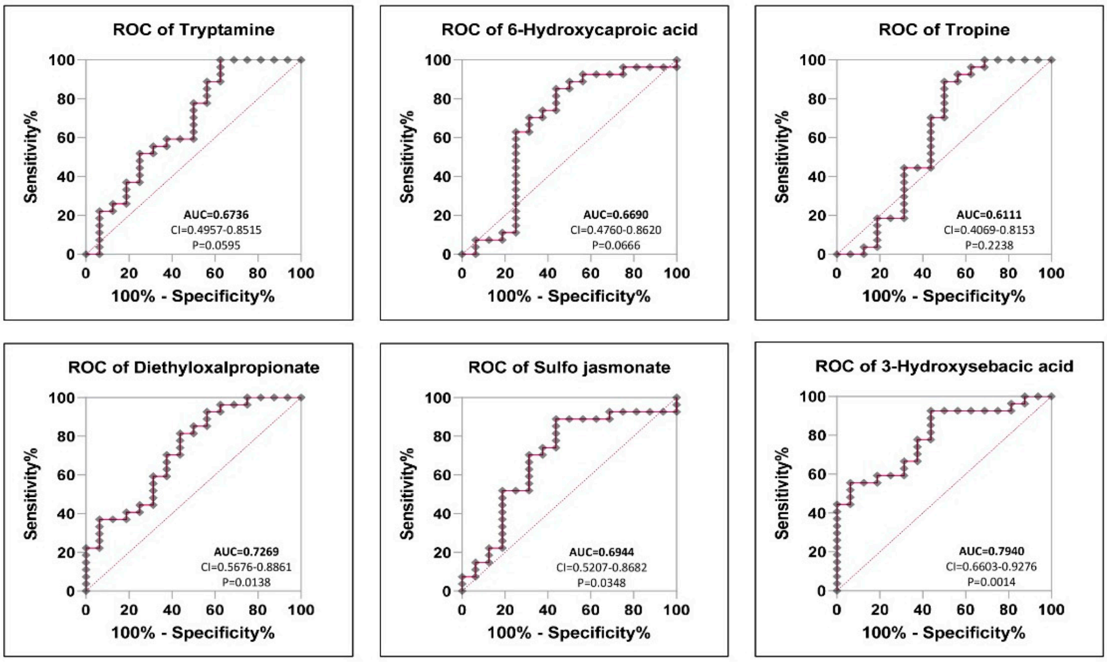

ROC curves for the metabolites shown in Supplementary Figure 3 (Tryptamine, 6-hydroxycaproic acid, tropine, diethyloxalproprionate, sulfo jasmonate, 3-hydroxysebacic acid). Area under the ROC curve (AUC) is calculated as % sensitivity vs 100-% specificity. AUC>0.85 is highly discriminatory. Note that although the six metabolites are differentially expressed in Urothelial Carcinoma vs Control, they were unable to discriminate between the two conditions.

Supplementary Table S1

Functional and pathway analysis of metabolites that are differentially expressed between Urothelial Carcinoma and Control groups, after adjusting for age and sex (adjusted p-value <0.05; -1.5≥fold change≥1.5)

Platform	BinBase name	Known Origin/Function	Pathway
Primary metabolites (GCTOF MS)	glycerol-3-galactoside		Galactose metabolism; Glycerolipid metabolism
	hippuric acid	Increased with consumption of phenolic compounds including whole grain, exposure to toluene; associated with phenylketonuria, propionic acidemia, and tyrosinemia I	Phenylalanine metabolism
Secondary and charged metabolites (HILIC-QTOF MS)	E-4031	Experimental class III antiarrhythmic drug that blocks potassium channels of the hERG-type	Potassium channel blocker
	Riluzole	Glutamate blocker that can treat amyotrophic lateral sclerosis	Neuroactive ligand-receptor interaction; Glutamatergic synapse; Dopaminergic synapse
	Diethyloxalpropionate	The progression of inflammation	Cysteine and methionine metabolism; Sulfur metabolism
	3-Methyl-2-oxovaleric acid	Neurotoxin, acidogen, and metabotoxin, associated with maple syrup urine disease	Valine, leucine and isoleucine degradation; Valine, leucine and isoleucine biosynthesis; Glucosinolate biosynthesis; 2-Oxocarboxylic acid metabolism
	Threonine	Amino acid	Glycine, serine and threonine metabolism; Monobactam biosynthesis; Valine, leucine and isoleucine biosynthesis; Porphyrin and chlorophyll metabolism; Aminoacyl-tRNA biosynthesis
	N-Acetylphenylalanine	Metabolite of phenylalanine upregulated in urine of patients with phenylketonuria	Phenylalanine metabolism
	3,4-Dihydroxyphenylglycol	Catechol involved in tyrosinemia and menkes disease. Also found in olives	Tyrosine metabolism
	N-alpha-methylhistamine	Aralkylamines	Histidine metabolism
	3-Hydroxysebacic acid	Urinary 3-hydroxydicarboxylic acid metabolite elevated in patients with peroxisomal disorders such as Zellweger syndrome and in malnourished infants	

5-Methoxypsoralen	Found in anises, figs, and parsnips	Biosynthesis of phenylpropanoids
6-Hydroxycaproic acid	Manufacture of its esters for artificial flavors; Utilization for growth	Fatty acyls
Octanoylcarnitine	Upregulated in medium-chain acyl-CoA dehydrogenase (MCAD) deficiency characterized by an intolerance to prolonged fasting, recurrent episodes of hypoglycemic coma; associated with celiac disease and glutaric aciduria II	Fatty acyls
Otenzepad	Muscarinic M2 receptor antagonist	
2-Hydroxy-4-methylpentanoic acid	End product of leucine metabolism in muscle and connective tissue; improve muscle recovery; upregulated in patients with short-bowel syndrome, and in maple syrup urine disease; indicator of oxidative stress; metabolite of Lactobacillus and fungus.	Valine, leucine and isoleucine biosynthesis; 2-Oxocarboxylic acid metabolism
Taxifolin	Antioxidant; Flavanonol	Flavonoid biosynthesis; Biosynthesis of phenylpropanoids
Sulfo jasmonate	Derived from α-linolenic acid (18:3)	Jasmonate Biochemical Pathway
Tryptamine	Catabolite of tryptophan converted by the gut microbiota; precursor to hormones and neurotransmitters.	Tryptophan metabolism; Indole alkaloid biosynthesis; Neuroactive ligand-receptor interaction
Flunitrazepam	Benzodiazepine used to treat severe insomnia and assist with anesthesia	Neuropsychiatric agent; Benzodiazepine sedative-hypnotics; GABA-A receptor agonist
Sarcosine	N-methyl derivative of glycine, found in muscles and other body tissues.	Glycine, serine and threonine metabolism; Arginine and proline metabolism
Cocaethylene A	Recreational drug with stimulant, euphoriant, anorectic, sympathomimetic and local anesthetic properties	Tropane, piperidine and pyridine alkaloid biosynthesis; Biosynthesis of alkaloids derived from ornithine, lysine and nicotinic acid
Propofol .beta.-D-glucuronide	Metabolite of propofol, a short-acting general anesthetic agent	Pentose and glucuronate interconversions; Ascorbate and aldarate metabolism; Bile secretion
N-Acetyl-S-benzyl-L-cysteine	Metabolite of toluene used in the diagnosis of toluene exposure	Glutathione metabolism
Olopatadine	Antihistamine	Rhodopsin family
Convolvamine	Tropane alkaloid that occurs in plants such as potato and tomato	Phytochemical Compounds

Tropine	Derivative of tropane and a central building block of many chemicals active in the nervous system, including tropane alkaloids	Tropane, piperidine and pyridine alkaloid biosynthesis; Biosynthesis of alkaloids derived from ornithine, lysine and nicotinic acid

Supplementary Table S2

Comparison of primary metabolites and biological amines from the urine of male and female dogs that show significant differences in the Urothelial Carcinoma vs Control arms. Highlighted are the ones that are also included in Table 2 (all subjects). The metabolites identified as discriminatory in all subjects are bolded as well. Note that only metabolites that showed a log -1.5≥fold change≥1.5 and adjusted p-value<0.05 are shown.

	MALE			FEMALE		
**Metabolite name**	**logFC**	**P.Value**	**adj.P.Val**	**logFC**	**P.Value**	**adj.P.Val**
2'-Deoxyadenosine	-2.02435	0.000152	0.008977	-2.2269	9.65E-05	0.006444
3-Methyl-2-oxovaleric acid	1.92276	8.57E-05	0.00724	2.056688	7.51E-05	0.00634
4-Acetylbutyric acid	3.016982	0.000308	0.015156	2.978025	0.000674	0.028458
5-Methoxypsoralen	-2.77674	1.69E-05	0.001669	-3.02639	1.22E-05	0.001607
Arginine				1.953384	0.001621	0.045624
Cocaethylene A	-2.80975	0.000269	0.014469	-3.07369	0.000194	0.010441
Convolvamine	-4.61692	1.71E-07	0.000101	-4.72357	3.25E-07	0.000192
Cystathionine	-3.32658	0.001302	0.042754	-3.49229	0.001432	0.042653
E-4031	6.883452	1.07E-05	0.0016	7.083815	1.41E-05	0.001607
Guanine	-2.10924	0.00037	0.016809	-2.24752	0.00036	0.017741
**hippuric acid**	**-4.47555**	**1.46E-07**	**2.98E-05**	**-4.5892**	**2.96E-07**	**6.03E-05**
N-Acetyl-S-benzyl-L-cysteine	-4.81061	1.08E-05	0.0016	-5.02082	1.37E-05	0.001607
**N-alpha-methylhistamine**	**-1.80922**	**0.000116**	**0.008549**	**-1.84066**	**0.000192**	**0.010441**
Phenelzine	-1.53166	0.000787	0.030149	-1.53383	0.001394	0.042653
Phenylacetylglycine				1.814252	0.00109	0.037891
Propofol .beta.-D-glucuronide	-3.96961	0.0004	0.016901	-4.2011	0.000409	0.018581
**Sarcosine**	**-2.89538**	**0.00015**	**0.008977**	**-3.18448**	**9.81E-05**	**0.006444**
Sulfo jasmonate	-2.89812	0.001532	0.047659	-3.08881	0.001443	0.042653
Taxifolin	-2.64386	0.000951	0.033045	-2.8525	0.000785	0.030168
Threonine	2.28876	0.000816	0.030149	2.422169	0.000817	0.030168
Tropine	-5.20554	1.47E-05	0.001669	-5.47283	1.63E-05	0.001607
Tryptamine	-4.01307	1.72E-06	0.000507	-4.27497	1.53E-06	0.000453

Supplementary Table S3

Differences in metabolites from the urine of retriever vs non-retriever in the Urothelial Carcinoma and Control arms.

RETRIEVER VS NON-RETRIEVER								
	UC				U/UTI			
**Metabolite name**	**logFC**	**AveExpr**	**P.Value**	**adj.P.Val**	**logFC**	**AveExpr**	**P.Value**	**adj.P.Val**
Acetominophen	5.515194	10.08659	9.69E-06	0.002498	5.561471	10.08659	9.84E-06	0.003077
Tetradecanedioic acid	9.038849	10.66414	8.99E-06	0.002498	8.985079	10.66414	1.14E-05	0.003077
1,11-Undecanedicarboxylic acid	6.276369	11.51583	1.27E-05	0.002498	6.249875	11.51583	1.56E-05	0.003077
4-Methylcatechol	3.465373	10.00231	2.74E-05	0.003245	3.481306	10.00231	2.92E-05	0.004074
Pinacidil	6.225059	8.937388	2.69E-05	0.003245	6.180513	8.937388	3.45E-05	0.004074
Lipoic acid	5.812235	7.914433	5.29E-05	0.005211	5.825389	7.914433	5.95E-05	0.005861
3-Hydroxypropanoic acid	2.86199	11.70459	0.000147	0.012426	2.839741	11.70459	0.000182	0.01534
alpha-Hydroxyhippuric acid	2.663976	8.463497	0.000189	0.013981	2.658843	8.463497	0.000219	0.015683
Butyrylglycine	3.003771	8.919083	0.000261	0.017136	3.055047	8.919083	0.000239	0.015683
Vanillic acid	6.497938	14.4596	0.000308	0.018211	6.497604	14.4596	0.000344	0.020328
Adipic acid	2.551225	10.93456	0.000504	0.027068	2.551884	10.93456	0.000552	0.029684
Cinnamic acid	3.175433	7.991106	0.000659	0.032449	3.217905	7.991106	0.000644	0.031711
Valsartan	2.286284	8.173027	0.000834	0.037898	2.269031	8.173027	0.001004	0.045641

Supplementary Table S4

Comparison of primary metabolites and biological amines from the urine of terriers only that show significant differences in the Urothelial Carcinoma (UC) vs Control arms. The list includes primary metabolites, secondary amines as well as lipids. Only metabolites that showed a log -1.5≥fold change≥1.5 and adjusted p-value<0.05 are shown. Highlighted are the ones that are common with the ones showing overall differences between UC vs Control.

	TERRIER			
**Metabolite name**	**logFC**	**AveExpr**	**P.Value**	**adj.P.Val**
5-Methoxypsoralen	-2.8969	6.592871	1.09E-05	0.00329
Valdecoxib	-2.7606	7.433488	1.11E-05	0.00329
Convolvamine	-2.84725	8.340094	7.02E-05	0.013839
Sarcosine	-2.62297	7.025748	0.000216	0.031947

Supplementary Table S5

Comparison of primary metabolites and biological amines from the urine of retrievers only that show significant differences in the Urothelial Carcinoma (UC) vs Control arms. The list includes primary metabolites, secondary amines as well as lipids. Only metabolites that showed a log -1.5≥fold change≥1.5 and adjusted p-value<0.05 are shown. Highlighted are the ones that are common with the ones showing overall differences between UC vs control.

	RETRIEVERS			ALL OTHER BREEDS		
**Metabolite name**	**logFC**	**P.Value**	**adj.P.Val**	**logFC**	**P.Value**	**adj.P.Val**
E-4031	4.97	0.0004	0.0109	5.28	0.0004	0.0115
2,3-Dihydroxybenzoic acid B	3.15	0.0003	0.0099	3.29	0.0004	0.0115
4-Acetylbutyric acid	2.83	0.0001	0.0047	2.80	0.0003	0.0098
FA 26:0 (cerotic acid)	2.82	0.0007	0.0274	2.89	0.0011	0.0452
FA 24:0 (lignoceric acid)	2.79	0.0004	0.0234	2.89	0.0006	0.0349
Riluzole	2.40	0.0006	0.0157	2.68	0.0004	0.0115
FA 23:0;	2.37	0.0003	0.0234	2.47	0.0005	0.0339
Threonine	2.16	0.0002	0.0064	2.29	0.0002	0.0078
FA 24:1 (nervonic acid)	2.08	0.0000	0.0095	2.18	0.0000	0.0135
1-kestose	1.88	0.0007	0.0494			
N-Acetylphenylalanine	1.80	0.0001	0.0047	1.88	0.0002	0.0065
Diethyloxalpropionate	1.75	0.0024	0.0410	1.90	0.0021	0.0387
Salicylic alcohol	1.74	0.0035	0.0471	1.90	0.0029	0.0444
3-Methyl-2-oxovaleric acid	1.61	0.0001	0.0047	1.74	0.0001	0.0053
2'-Deoxyadenosine	-1.57	0.0009	0.0237	-1.75	0.0006	0.0165
Octanoylcarnitine	-1.59	0.0027	0.0418			
Metanephrine	-1.62	0.0035	0.0471	1.64	0.0003	0.0096
Phenylacetylglycine				1.64	0.0008	0.0188
2-[(4-Aminobenzoyl)amino]acetic acid				1.57	0.0031	0.0452
Hypoxanthine				-1.60	0.0002	0.0178
Cer d42:2 Isomer A	-1.64	0.0005	0.0247	-1.65	0.0010	0.0452
N-alpha-methylhistamine	-1.65	0.0001	0.0045	-1.68	0.0001	0.0065
D-Glucosamine	-1.68	0.0021	0.0389	-1.75	0.0029	0.0444
Homoarginine	-1.78	0.0001	0.0059	-1.96	0.0001	0.0055
3-Hydroxysebacic acid	-1.81	0.0011	0.0259	-1.97	0.0009	0.0205
5-Methoxypsoralen	-1.86	0.0011	0.0268	-2.05	0.0009	0.0205
Diazoxide	-1.91	0.0019	0.0359	-1.96	0.0029	0.0444
6-Hydroxycaproic acid	-1.92	0.0018	0.0359	-2.27	0.0007	0.0174
Otenzepad	-2.08	0.0018	0.0359	-2.24	0.0019	0.0358
2-Hydroxy-4-methylpentanoic acid	-2.12	0.0022	0.0389	-2.46	0.0011	0.0222
L-Saccharopine	-2.19	0.0016	0.0358	-2.43	0.0011	0.0228
Cer d36:1	-2.20	0.0003	0.0234	-2.26	0.0005	0.0339
TAG 56:4; TAG 18:0-18:2-20:2;	-2.32	0.0002	0.0234	-2.58	0.0001	0.0212
Haloperidol	-2.64	0.0024	0.0414	-2.81	0.0027	0.0444
Taxifolin	-2.84	0.0000	0.0011	-3.07	0.0000	0.0011
Sulfo jasmonate	-2.96	0.0002	0.0070	-3.15	0.0002	0.0080
Sarcosine	-3.00	0.0000	0.0004	-3.29	0.0000	0.0003

Flunitrazepam	-3.11	0.0000	0.0022	-3.38	0.0000	0.0022
Cer-NS d34:1; Cer-NS d18:1/16:0;	-3.24	0.0004	0.0234	-3.43	0.0005	0.0339
Cocaethylene A	-3.27	0.0000	0.0001	-3.58	0.0000	0.0001
Tryptamine	-3.64	0.0000	0.0000	-3.90	0.0000	0.0000
Olopatadine	-4.00	0.0001	0.0047	-4.35	0.0001	0.0053
hippuric acid	-4.02	0.0000	0.0001	-4.14	0.0000	0.0001
Convolvamine	-4.05	0.0000	0.0001	-4.15	0.0000	0.0001
Propofol .beta.-D-glucuronide	-4.09	0.0000	0.0011	-4.38	0.0000	0.0012
1'-Hydroxymidazolam .beta.-D-glucuronide	-4.40	0.0033	0.0467	-4.90	0.0028	0.0444
N-Acetyl-S-benzyl-L-cysteine	-4.47	0.0000	0.0003	-4.70	0.0000	0.0004
Tropine	-4.92	0.0000	0.0003	-5.22	0.0000	0.0003

Supplementary Table S6

Comparison of primary metabolites and biological amines from the urine of dogs with Urothelial Carcinoma who had been exposed to antibiotics prior to urine collection vs those who had not.

Metabolite name	logFC	AveExpr	P.Value	adj.P.Val	HMDB Information
Tetradecanedioic acid	9.117359	10.66414	1.45E-05	0.002854	Usually ingested
Vanillic acid	6.578139	14.4596	0.000351	0.018841	phenolic acid found in some forms of vanilla and many other plant extracts. found in the urine of humans who have consumed coffee, chocolate, tea, and vanilla-flavoured confectionary. selectively and specifically inhibits 5'nucleotidase activity. microbial metabolite found in Amycolatopsis, Delftia, and Pseudomonas.
1,11-Undecanedicarboxylic acid	6.279259	11.51583	1.32E-05	0.002854	Usually ingested
Pinacidil	6.241122	8.937388	2.18E-05	0.003176	not a naturally occurring metabolite.
Lipoic acid	5.788047	7.914433	4.47E-05	0.004403	vitamin-like antioxidant that acts as a free-radical scavenger. Pathways - Nitrogen metabolism, Glycine, serine and threonine metabolism, Dimethylglycine Dehydrogenase Deficiency, Dihydropyrimidine Dehydrogenase Deficiency (DHPD), Sarcosinemia, Hyperglycinemia.
Acetominophen	5.472752	10.08659	8.81E-06	0.002854	analgesic. Ingested.
4-Methylcatechol	3.459723	10.00231	2.69E-05	0.003176	coffee and cocoa powders and in a lower concentration in beers. metabolite of homoprotocatechuic acid. known to induce the production of brain-derived neurotrophic factor (BDNF). Usually ingested.
Cinnamic acid	3.161086	7.991106	0.000662	0.032616	obtained from oil of cinnamon, or from balsams such as storax. exists in all living organisms, ranging from bacteria to plants to humans. Ingested.
Butyrylglycine	3.010225	8.919083	0.000259	0.015282	disorders associated with mitochondrial fatty acid beta-oxidation
3-Hydroxypropanoic acid	2.874523	11.70459	0.000159	0.013458	Propanoate metabolism pathway, ingested in food
alpha-Hydroxyhippuric acid	2.652321	8.463497	0.000213	0.01488	derivative of hippuric acid, usually ingested in food.
Adipic acid	2.595833	10.93456	0.000227	0.01488	used mainly in the production of nylon, controlled-release formulation matrix tablets. good biomarker of jello consumption. certain disorders (such as diabetes and glutaric aciduria type I) can lead to elevated levels of adipic acid in urine. associated with 3-hydroxy-3-methylglutaryl-CoA lyase deficiency, carnitine-acylcarnitine translocase deficiency, malonyl-Coa decarboxylase deficiency, and medium Chain acyl-CoA dehydrogenase deficiency, which are inborn errors of metabolism. Adipic acid is also microbial metabolite found in Escherichia.
Valsartan	2.296565	8.173027	0.00076	0.034546	angiotensin-receptor blocker used to treat a variety of cardiac conditions including hypertension, diabetic nephropathy and heart failure.
